# Crystal structure of bis­(2-amino­anilinium) hydrogen phosphate

**DOI:** 10.1107/S2056989016004709

**Published:** 2016-03-22

**Authors:** Reena Ittyachan, Melesuparambil Sundaram Ahigna, Rajamony Jagan

**Affiliations:** a Department of Physics, Sacred Heart College, Chalakudy, Kerala 680 307, India; b Department of Chemistry, Indian Institute of Technology Madras, Chennai 600 036, India

**Keywords:** crystal structure, 2-amino­anilinium, hydrogen phosphate, supra­molecular network, hydrogen bonds

## Abstract

The asymmetric unit of the title compound, 2C_6_H_9_N_2_^+^·HPO_4_^2−^, comprises two 2-amino­anilinium cations and one hydrogen phosphate dianion. In the crystal, the HPO_4_^2−^ dianions are linked by O—H⋯O hydrogen bonds into chains along [100]. The inorganic anionic chains and organic cations are linked by N—H⋯O and N—H⋯N hydrogen bonds, forming a two-dimensional supra­molecular network extending parallel to (001).

## Abstract 2

In the title compound, the hydrogen phosphate anions are linked by O—H⋯O hydrogen bonds into chains parallel to [100]. The inorganic anionic chains and the organic cations are linked by N—H⋯O and N—H⋯N hydrogen bonds, forming a two-dimensional supra­molecular network extending parallel to (001).

## Chemical context   

Inorganic–organic hybrid compounds are of current inter­est due to their fascinating architectures and potential applications in crystal engineering and supra­molecular chemistry (Singh *et al.*, 2011 ▸; Direm *et al.*, 2015 ▸). Among the explored hybrid compounds, organic phosphates formed as a result of the reaction with inorganic oxy acids such as ortho­phospho­ric acid (H_3_PO_4_) and organic amines and amides are particularly inter­esting. Organic mono­hydrogen (HPO_4_^2−^) and di­hydrogen phosphate (H_2_PO_4_^−^) compounds provide a class of materials with numerous practical and potential uses in various fields such as biomolecular sciences, catalysis, liquid-crystal-material development, ferroelectrics, non-linear optical and supra­molecular studies (Khan *et al.*, 2009 ▸; Evans *et al.*, 2008 ▸; Balamurugan *et al.*, 2010 ▸). Non-covalent inter­actions, such as hydrogen bonding and other weak inter­actions, represent the basic set of tools for the construction of elabor­ate supra­molecular architectures of organic or inorganic–organic compounds. In this respect, the potential of mono­hydrogen and di­hydrogen phosphate anions as useful building blocks has been investigated structurally (Shylaja *et al.*, 2008 ▸; Oueslati *et al.*, 2007 ▸; Jagan *et al.*, 2015 ▸; Trojette *et al.*, 1998 ▸; Soumhi & Jouini, 1995 ▸). Here we report the structure and the self-assembled supra­molecular architecture exhibited through the formation of O—H⋯O, N—H⋯O and N—H⋯N hydrogen bonds in bis­(2-amino­anilinium) hydrogen phosphate.
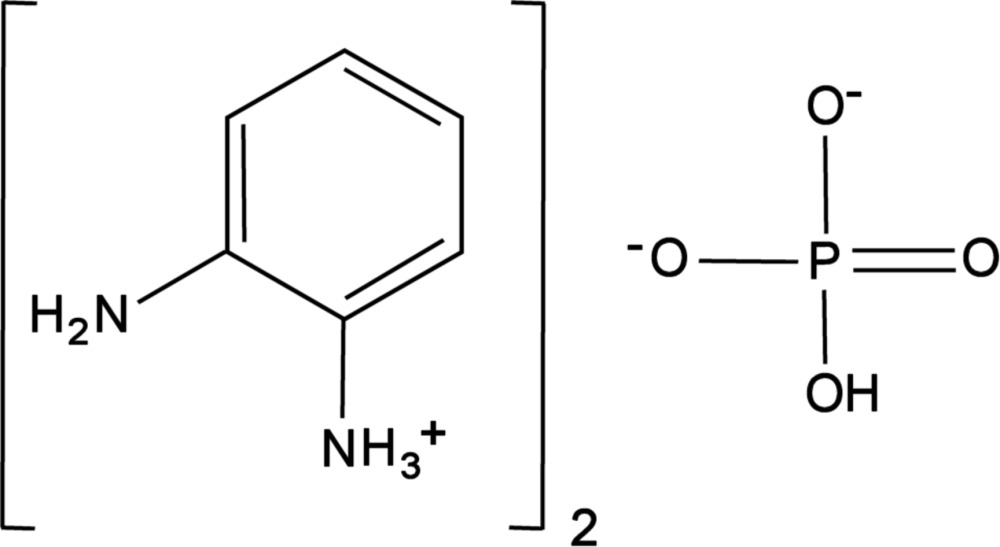


## Structural commentary   

**Figure 1:**
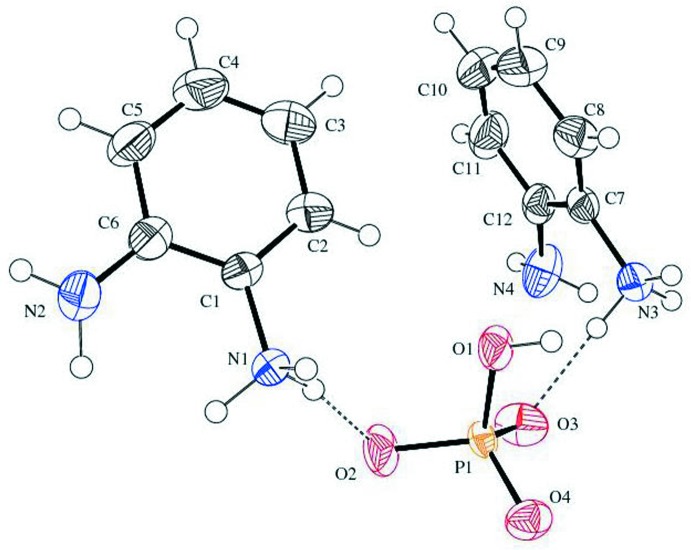
The asymmetric unit of the title compound with displacement ellipsoid drawn at the 40% probability level. The dashed lines represent hydrogen bonds.

The asymmetric unit of the title compound comprises two 2-amino­anilinium cations and one hydrogen phosphate dianion (Fig. 1 ▸). The existence of the hydrogen phosphate anion is confirmed by the P—O bond distances, and the presence of a relevant density peak at a distance from the oxygen atom O1 confirms the hydroxyl group of the anion. The bond distance P1—O1 = 1.561 (2) Å indicates single-bond character, while the bond distances P1—O2 = 1.504 (2), P1—O3 = 1.504 (2) and P1—O4 = 1.497 (2) Å reveal the resonating P—O bonds of the hydrogen phosphate anion. As expected (Rao *et al.*, 2010 ▸; Peng & Zhao, 2010 ▸), in both cations the C—N bond [C1—N1 = 1.450 (3), C7—N3 = 1.450 (4) Å] involving the ammonium group is longer than that in the amine group [C6—N2 = 1.384 (4), C12—N4 = 1.383 (4) Å]. The phenyl rings of the *o*-phenyl­enedi­ammonium cations are almost perpendicular to one another [dihedral angle 86.53 (2)°].

## Supra­molecular features   

**Figure 2:**
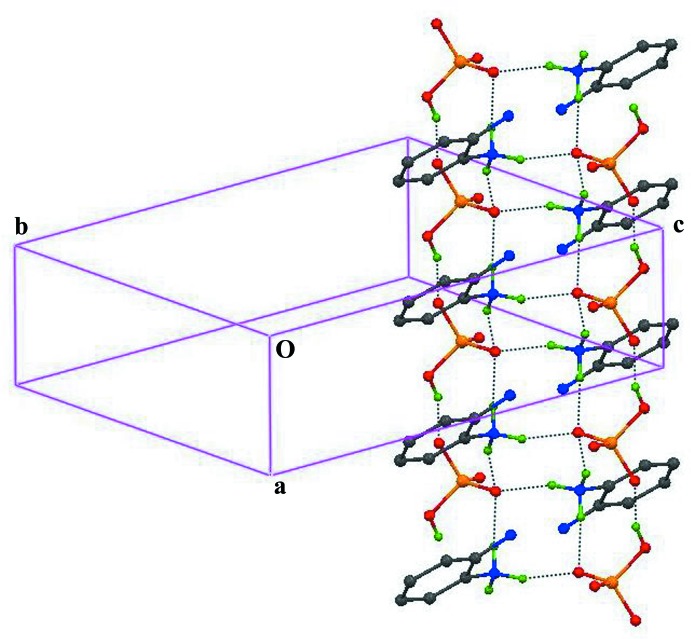
Partial packing diagram of the title compound showing the formation of an organic–inorganic supra­molecular ladder through N—H⋯O and O—H⋯O hydrogen bonds extending along [100]. The formation of rings with 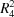
(8) and 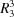
(10) graph-set motifs is also shown. Hydrogen atoms not involved in hydrogen bonding are omitted for clarity.

**Figure 3:**
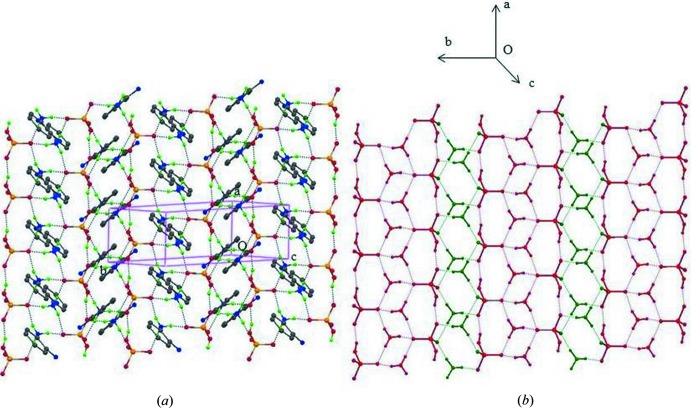
Crystal packing of the title compound showing (*a*) the formation through hydrogen bonds (dashed lines) of an organic–inorganic supra­molecular sheet extending parallel to (001) and (*b*) the (001) network in which red represents the [100] ladder, bridged by the cations (represented in green) through N—H⋯O hydrogen bonds.

**Figure 4:**
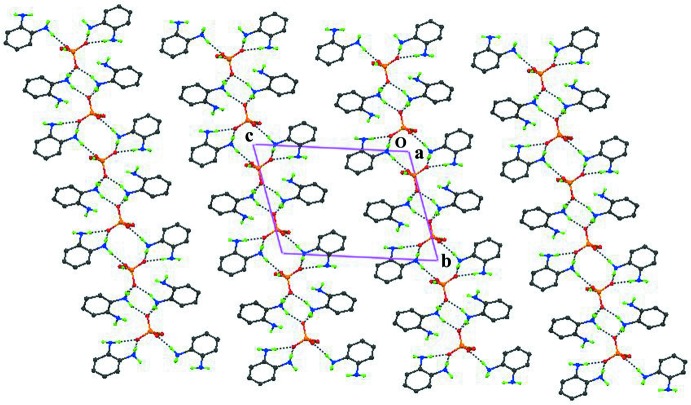
Packing of the title compound, viewed down the *a* axis, showing the arrangement of the (001) two-dimensional supra­molecular networks stacked along the *c* axis. Dashed lines indicate hydrogen bonds.

In the title structure, the hydrogen phosphate anion and 2-amino­anilinium cations possess a number of donor and acceptor sites, which leads to the formation of a variety of hydrogen bonds *viz*. O—H⋯O, N—H⋯O and N—H⋯N (Table 1 ▸). The O1—H1*D*⋯O2^i^ hydrogen bond [symmetry code: (i) *x* + 1, *y*, *z*] connects adjacent hydrogen phosphate anions, forming anionic chains extending along [100]. The oxygen atom O3 acts as a trifurcated hydrogen-bond acceptor for the donor nitro­gen atom N1 at (*x*, *y*, *z*), (−1 + *x*, *y*, *z*) and (1 − *x*, 1 − *y*, 2 − *z*), forming a one-dimensional supra­molecular ladder extending along [100] as shown in Fig. 2 ▸. In the ladder, centrosymmetrically related anions and cations are inter­linked through N3—H3*C*⋯O3, N3—H3*A*⋯O3^i^ and N3—H3*B*⋯O3^iv^ [symmetry code: (iv) −*x* + 1, −*y* + 1, −*z* + 2] hydrogen bonds, forming two types of fused rings of 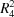
(8) graph-set motif. The association of O—H⋯O hydrogen bonds in the anionic chains with the N—H⋯O hydrogen bonds in the ladder forms heteromeric 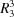
(10) hydrogen-bonded motifs. Adjacent ladders are further bridged by N1—H1*B*⋯O2, N1—H1*A*⋯O4^ii^ and N1—H1*C*⋯O4^iii^ [symmetry codes: (ii) −*x* + 1, −*y* + 2, −*z* + 2; (iii) −*x*, −*y* + 2, −*z* + 2] hydrogen bonds, resulting in the formation of a two-dimensional organic–inorganic supra­molecular layered network parallel to (001) (Fig. 3 ▸). In the (001) network, the bridging cations make rings of 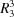
(10) and 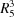
(12) motifs through the three charge-assisted N—H⋯O and the O1—H1*D*⋯O2^i^ hydrogen bonds. In addition, the N2—H2*A*⋯O4^iii^, N1—H1*C*⋯O4^iii^ and N4—H4*B*⋯N2^v^ [symmetry codes: (iii) −*x*, −*y* + 2, −*z* + 2; (v) *x*, −1 + *y*, *z*] hydrogen bonds stabilize the (001) network. In the crystal structure (Fig. 4 ▸), adjacent organic–inorganic layers are separated by a distance equal to the length of the *c* axis.

## Database Survey   

A CSD database search (*ConQuest* 1.17; Groom & Allen, 2014 ▸) showed 48 entries for hydrogen phosphate salts formed with various amino cations. It is inter­esting to observe that most of the reported structures of hydrogen phosphate salts are hydrated (33 structures) compared to the reported structures of di­hydrogen phosphate and phosphate salts. Most of the hydrogen phosphate structures reported contain alkyl cations (Ilioudis *et al.*, 2002 ▸; Mrad *et al.*, 2012 ▸; Li *et al.*, 2010 ▸), in which the alkyl cations encapsulated between chains of hydrogen phosphate are flexible with respect to the nature of the cations, which may induce a change in physical properties (Baouab & Jouini, 1998 ▸). As observed in the title compound, in the crystal structure of 2-amino­anilinium di­hydrogen phosphate (CSD refcode: SAYWAQ; Trojette *et al.*, 1998 ▸), the di­hydrogen phosphate anions form chains, which are bridged by 2-amino­anilinium cations through N—H⋯O hydrogen bonds, generating a two-dimensional inorganic–organic network. Conversely, in the crystal structure of 1,2-phenyl­enedi­ammonium bis­(di­hydrogen phosphate) (ZAYPAQ; Soumhi & Jouini, 1995 ▸), the anions form inorganic sheets inter­linked by 1,2-phenyl­enedi­ammonium cations, thus generating a three-dimensional inorganic–organic framework. This can be attributed to the double protonation of the cations in ZAYPAQ compared to the title compound and SAYWAQ. In the crystal structure of 2-amino­anilinium perchlorate monohydrate (KAJGUY; Raghavaiah *et al.*, 2005 ▸), the 2-amino­anilinium cation, the perchlorate anion and the lattice water mol­ecule assemble into a unique hydrogen-bonded supra­molecular framework, forming alternate hydro­phobic and hydro­philic zones.

## Synthesis and crystallization   

The title compound was prepared by dissolving in water *o*-phenyl­enedi­amine and ortho­phospho­ric acid in a 2:1 molar ratio. The resulting mixture was stirred continuously for 3 h with constant heating maintained at 333 K. The solution was then cooled, filtered and kept for crystallization without any disturbance. Good diffraction-quality crystals were obtained after one week.

## Refinement   

Crystal data, data collection and structure refinement details are summarized in Table 2 ▸. The hydrogen atoms associated with the N and O atoms were localized in a difference electron-density map and refined with the N—H and O—H distances constrained to values of 0.90 (2) and 0.85 (1) Å, respectively. All other hydrogen atoms were placed in calculated positions and allowed to ride on their parent atoms, with C—H = 0.93 Å and *U*_iso_(H) = 1.2*U*_eq_(C).

## Supplementary Material

Crystal structure: contains datablock(s) I, global. DOI: 10.1107/S2056989016004709/rz5186sup1.cif

Structure factors: contains datablock(s) I. DOI: 10.1107/S2056989016004709/rz5186Isup2.hkl

Supporting information file. DOI: 10.1107/S2056989016004709/rz5186Isup3.cml

CCDC reference: 1469440

Additional supporting information:  crystallographic information; 3D view; checkCIF report

## Figures and Tables

**Table 1:** Hydrogen-bond geometry (Å, °)

*D*—H⋯*A*	*D*—H	H⋯*A*	*D*⋯*A*	*D*—H⋯*A*
O1—H1*D*⋯O2^i^	0.85 (1)	1.65 (1)	2.470 (3)	164 (4)
N3—H3*A*⋯O3^i^	0.90 (2)	2.06 (2)	2.928 (3)	160 (3)
N1—H1*A*⋯O4^ii^	0.92 (2)	1.81 (2)	2.720 (3)	171 (3)
N1—H1*C*⋯O4^iii^	0.93 (2)	2.02 (2)	2.953 (3)	179 (3)
N2—H2*A*⋯O4^iii^	0.92 (2)	1.99 (2)	2.904 (4)	170 (3)
N4—H4*A*⋯O4^iv^	0.88 (2)	2.45 (3)	3.188 (4)	142 (3)
N3—H3*B*⋯O3^iv^	0.91 (2)	1.87 (2)	2.740 (3)	159 (3)
N3—H3*C*⋯O3	0.91 (2)	1.87 (2)	2.778 (3)	176 (3)
N1—H1*B*⋯O2	0.92 (2)	1.83 (2)	2.734 (3)	169 (3)
N4—H4*B*⋯N2^v^	0.89 (2)	2.33 (2)	3.210 (4)	172 (3)

Symmetry codes: (i) 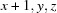; (ii) 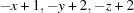; (iii) 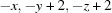; (iv) 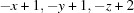; (v) 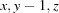.

**Table 2:** Experimental details

Crystal data	
Chemical formula	2C_6_H_9_N_2_^+^·HPO_4_^2−^
*M*_r_	314.28
Crystal system, space group	Triclinic, *P* 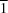
Temperature (K)	296
*a*, *b*, *c* (Å)	4.7613 (7), 10.8925 (17), 15.054 (2)
α, β, γ (°)	107.263 (3), 94.060 (3), 94.549 (3)
*V* (Å^3^)	739.6 (2)
*Z*	2
Radiation type	Mo *K*α
μ (mm^−1^)	0.21
Crystal size (mm)	0.30 × 0.20 × 0.20

Data collection	
Diffractometer	Bruker Kappa APEXII CCD Diffractometer
Absorption correction	Multi-scan (*SADABS*; Bruker, 2012 ▸)
*T*_min_, *T*_max_	0.865, 0.902
No. of measured, independent and observed [*I* > 2σ(*I*)] reflections	16948, 2841, 2271
*R*_int_	0.039
(sin θ/λ)_max_ (Å^−1^)	0.617

Refinement	
*R*[*F* ^2^ > 2σ(*F* ^2^)], *wR*(*F* ^2^), *S*	0.052, 0.119, 1.16
No. of reflections	2841
No. of parameters	242
No. of restraints	11
H-atom treatment	H atoms treated by a mixture of independent and constrained refinement
Δρ_max_, Δρ_min_ (e Å^−3^)	0.49, −0.34

Computer programs: *APEX2*, *SAINT* and *XPREP* (Bruker, 2012 ▸), *SHELXS2014* (Sheldrick, 2008 ▸), *SHELXL2014* (Sheldrick, 2015 ▸), *ORTEP-3 for Windows* (Farrugia, 2012 ▸), *Mercury* (Macrae *et al.*, 2008 ▸) and *PLATON* (Spek, 2009 ▸).

## supplementary crystallographic information

### Crystal data

**Table:**

2C_6_H_9_N_2_^+^·HPO_4_^2^^−^	*Z* = 2
*M**_r_* = 314.28	*F*(000) = 332
Triclinic, *P*1	*D*_x_ = 1.411 Mg m^−^^3^
*a* = 4.7613 (7) Å	Mo *K*α radiation, λ = 0.71073 Å
*b* = 10.8925 (17) Å	Cell parameters from 7191 reflections
*c* = 15.054 (2) Å	θ = 2.8–26.1°
α = 107.263 (3)°	µ = 0.21 mm^−^^1^
β = 94.060 (3)°	*T* = 296 K
γ = 94.549 (3)°	Block, brown
*V* = 739.6 (2) Å^3^	0.30 × 0.20 × 0.20 mm

### Data collection

**Table:**

Bruker Kappa APEXII CCD Diffractometer	2271 reflections with *I* > 2σ(*I*)
ω and φ scan	*R*_int_ = 0.039
Absorption correction: multi-scan (*SADABS*; Bruker, 2012)	θ_max_ = 26.0°, θ_min_ = 2.0°
*T*_min_ = 0.865, *T*_max_ = 0.902	*h* = −5→5
16948 measured reflections	*k* = −13→13
2841 independent reflections	*l* = −18→18

### Refinement

**Table:**

Refinement on *F*^2^	11 restraints
Least-squares matrix: full	H atoms treated by a mixture of independent and constrained refinement
*R*[*F*^2^ > 2σ(*F*^2^)] = 0.052	*w* = 1/[σ^2^(*F*_o_^2^) + (0.0318*P*)^2^ + 0.8089*P*] where *P* = (*F*_o_^2^ + 2*F*_c_^2^)/3
*wR*(*F*^2^) = 0.119	(Δ/σ)_max_ < 0.001
*S* = 1.16	Δρ_max_ = 0.49 e Å^−^^3^
2841 reflections	Δρ_min_ = −0.34 e Å^−^^3^
242 parameters	

### Special details

**Table:**

Geometry. All e.s.d.'s (except the e.s.d. in the dihedral angle between two l.s. planes) are estimated using the full covariance matrix. The cell e.s.d.'s are taken into account individually in the estimation of e.s.d.'s in distances, angles and torsion angles; correlations between e.s.d.'s in cell parameters are only used when they are defined by crystal symmetry. An approximate (isotropic) treatment of cell e.s.d.'s is used for estimating e.s.d.'s involving l.s. planes.

### Fractional atomic coordinates and isotropic or equivalent isotropic displacement parameters (Å^2^)

**Table:**

	*x*	*y*	*z*	*U*_iso_*/*U*_eq_	
C1	0.0716 (5)	0.9348 (3)	0.75677 (17)	0.0294 (6)	
C2	0.2315 (7)	0.8319 (3)	0.7246 (2)	0.0430 (7)	
H2	0.3277	0.7991	0.7669	0.042 (9)*	
C3	0.2477 (8)	0.7789 (4)	0.6308 (2)	0.0589 (10)	
H3	0.3532	0.7097	0.6090	0.068 (11)*	
C4	0.1069 (8)	0.8290 (4)	0.5698 (2)	0.0618 (10)	
H4	0.1203	0.7942	0.5061	0.082 (13)*	
C5	−0.0531 (7)	0.9289 (4)	0.6000 (2)	0.0546 (9)	
H5	−0.1503	0.9597	0.5566	0.059 (10)*	
C6	−0.0730 (6)	0.9859 (3)	0.6957 (2)	0.0375 (7)	
N1	0.0743 (5)	0.9908 (2)	0.85708 (15)	0.0290 (5)	
H1A	0.238 (5)	1.046 (3)	0.881 (2)	0.060 (10)*	
H1B	0.079 (6)	0.928 (2)	0.8862 (19)	0.039 (8)*	
H1C	−0.080 (5)	1.037 (3)	0.8734 (19)	0.040 (8)*	
N2	−0.2164 (6)	1.0939 (3)	0.7268 (2)	0.0492 (7)	
H2A	−0.300 (7)	1.106 (3)	0.7814 (17)	0.064 (11)*	
H2B	−0.337 (7)	1.100 (4)	0.680 (2)	0.071 (12)*	
C7	0.6532 (6)	0.4711 (3)	0.79919 (19)	0.0321 (6)	
C8	0.7961 (7)	0.5283 (3)	0.7439 (2)	0.0493 (8)	
H8	0.9487	0.5905	0.7699	0.049 (9)*	
C9	0.7133 (9)	0.4933 (4)	0.6491 (3)	0.0683 (11)	
H9	0.8085	0.5322	0.6110	0.076 (12)*	
C10	0.4896 (9)	0.4008 (4)	0.6120 (3)	0.0694 (12)	
H10	0.4328	0.3768	0.5483	0.071 (11)*	
C11	0.3490 (8)	0.3432 (3)	0.6677 (2)	0.0551 (9)	
H11	0.1989	0.2799	0.6410	0.061 (11)*	
C12	0.4257 (6)	0.3773 (3)	0.7630 (2)	0.0371 (7)	
N3	0.7285 (5)	0.5133 (2)	0.89942 (16)	0.0321 (5)	
H3A	0.895 (5)	0.564 (3)	0.917 (2)	0.052 (10)*	
H3B	0.745 (6)	0.449 (2)	0.9267 (19)	0.043 (9)*	
H3C	0.588 (5)	0.558 (3)	0.926 (2)	0.046 (9)*	
N4	0.2718 (6)	0.3266 (3)	0.8209 (2)	0.0508 (7)	
H4A	0.372 (7)	0.311 (4)	0.867 (2)	0.076 (13)*	
H4B	0.135 (6)	0.266 (3)	0.790 (2)	0.065 (11)*	
O1	0.5217 (4)	0.8037 (2)	0.92225 (14)	0.0429 (5)	
H1D	0.692 (3)	0.799 (3)	0.940 (2)	0.064 (11)*	
O2	0.0401 (4)	0.8199 (2)	0.95819 (16)	0.0524 (6)	
O3	0.2896 (5)	0.63962 (19)	0.98533 (15)	0.0475 (6)	
O4	0.4165 (4)	0.86357 (19)	1.08978 (13)	0.0407 (5)	
P1	0.31296 (13)	0.78084 (6)	0.99259 (5)	0.02434 (19)	

### Atomic displacement parameters (Å^2^)

**Table:**

	*U*^11^	*U*^22^	*U*^33^	*U*^12^	*U*^13^	*U*^23^
C1	0.0285 (14)	0.0316 (15)	0.0257 (13)	−0.0056 (11)	0.0010 (11)	0.0076 (11)
C2	0.0474 (18)	0.0450 (18)	0.0349 (16)	0.0065 (15)	0.0069 (14)	0.0084 (14)
C3	0.067 (2)	0.059 (2)	0.045 (2)	0.0181 (19)	0.0133 (17)	0.0015 (17)
C4	0.065 (2)	0.080 (3)	0.0335 (19)	0.011 (2)	0.0065 (16)	0.0061 (18)
C5	0.051 (2)	0.081 (3)	0.0347 (17)	0.0056 (19)	−0.0001 (15)	0.0248 (18)
C6	0.0325 (16)	0.0440 (17)	0.0370 (16)	−0.0026 (13)	0.0014 (12)	0.0161 (13)
N1	0.0293 (13)	0.0301 (13)	0.0267 (12)	0.0017 (10)	0.0011 (10)	0.0080 (10)
N2	0.0471 (17)	0.0611 (18)	0.0478 (17)	0.0145 (14)	0.0051 (14)	0.0270 (15)
C7	0.0321 (15)	0.0303 (15)	0.0350 (15)	0.0118 (12)	0.0032 (12)	0.0093 (12)
C8	0.0430 (19)	0.059 (2)	0.051 (2)	0.0094 (17)	0.0126 (15)	0.0215 (17)
C9	0.069 (3)	0.100 (3)	0.053 (2)	0.029 (2)	0.025 (2)	0.039 (2)
C10	0.075 (3)	0.098 (3)	0.035 (2)	0.034 (3)	0.0019 (19)	0.015 (2)
C11	0.060 (2)	0.053 (2)	0.0439 (19)	0.0135 (18)	−0.0113 (17)	0.0038 (16)
C12	0.0409 (17)	0.0309 (15)	0.0377 (16)	0.0121 (13)	−0.0036 (13)	0.0075 (12)
N3	0.0308 (14)	0.0290 (13)	0.0353 (13)	0.0021 (11)	0.0013 (10)	0.0083 (11)
N4	0.0484 (17)	0.0438 (17)	0.0583 (19)	−0.0114 (14)	−0.0164 (15)	0.0215 (15)
O1	0.0187 (10)	0.0748 (16)	0.0457 (12)	0.0028 (10)	0.0043 (9)	0.0346 (11)
O2	0.0195 (10)	0.0860 (18)	0.0641 (15)	0.0096 (10)	0.0061 (9)	0.0404 (13)
O3	0.0651 (15)	0.0264 (11)	0.0524 (13)	0.0050 (10)	0.0176 (11)	0.0115 (9)
O4	0.0401 (11)	0.0419 (12)	0.0329 (11)	−0.0036 (9)	0.0010 (9)	0.0031 (9)
P1	0.0160 (3)	0.0270 (4)	0.0319 (4)	0.0021 (2)	0.0026 (2)	0.0116 (3)

### Geometric parameters (Å, º)

**Table:**

C1—C6	1.380 (4)	C8—C9	1.382 (5)
C1—C2	1.391 (4)	C8—H8	0.9300
C1—N1	1.450 (3)	C9—C10	1.369 (6)
C2—C3	1.368 (4)	C9—H9	0.9300
C2—H2	0.9300	C10—C11	1.367 (5)
C3—C4	1.363 (5)	C10—H10	0.9300
C3—H3	0.9300	C11—C12	1.385 (4)
C4—C5	1.363 (5)	C11—H11	0.9300
C4—H4	0.9300	C12—N4	1.383 (4)
C5—C6	1.403 (4)	N3—H3A	0.902 (18)
C5—H5	0.9300	N3—H3B	0.913 (18)
C6—N2	1.384 (4)	N3—H3C	0.906 (18)
N1—H1A	0.923 (19)	N4—H4A	0.880 (19)
N1—H1B	0.915 (17)	N4—H4B	0.885 (19)
N1—H1C	0.931 (17)	O1—P1	1.561 (2)
N2—H2A	0.919 (18)	O1—H1D	0.846 (10)
N2—H2B	0.901 (19)	O2—P1	1.504 (2)
C7—C8	1.366 (4)	O3—P1	1.504 (2)
C7—C12	1.387 (4)	O4—P1	1.497 (2)
C7—N3	1.450 (4)		
			
C6—C1—C2	121.3 (3)	C7—C8—H8	120.1
C6—C1—N1	121.2 (2)	C9—C8—H8	120.1
C2—C1—N1	117.5 (2)	C10—C9—C8	119.3 (4)
C3—C2—C1	120.2 (3)	C10—C9—H9	120.4
C3—C2—H2	119.9	C8—C9—H9	120.4
C1—C2—H2	119.9	C11—C10—C9	120.6 (3)
C4—C3—C2	119.0 (3)	C11—C10—H10	119.7
C4—C3—H3	120.5	C9—C10—H10	119.7
C2—C3—H3	120.5	C10—C11—C12	121.3 (4)
C5—C4—C3	121.6 (3)	C10—C11—H11	119.3
C5—C4—H4	119.2	C12—C11—H11	119.3
C3—C4—H4	119.2	N4—C12—C11	121.7 (3)
C4—C5—C6	120.8 (3)	N4—C12—C7	121.0 (3)
C4—C5—H5	119.6	C11—C12—C7	117.1 (3)
C6—C5—H5	119.6	C7—N3—H3A	113 (2)
C1—C6—N2	121.9 (3)	C7—N3—H3B	116.0 (19)
C1—C6—C5	117.1 (3)	H3A—N3—H3B	105 (3)
N2—C6—C5	120.8 (3)	C7—N3—H3C	107 (2)
C1—N1—H1A	110 (2)	H3A—N3—H3C	110 (3)
C1—N1—H1B	110.7 (19)	H3B—N3—H3C	105 (3)
H1A—N1—H1B	105 (3)	C12—N4—H4A	116 (3)
C1—N1—H1C	112.3 (18)	C12—N4—H4B	113 (2)
H1A—N1—H1C	109 (3)	H4A—N4—H4B	116 (4)
H1B—N1—H1C	110 (3)	P1—O1—H1D	113 (2)
C6—N2—H2A	118 (2)	O4—P1—O2	111.69 (13)
C6—N2—H2B	110 (2)	O4—P1—O3	111.37 (12)
H2A—N2—H2B	112 (3)	O2—P1—O3	111.93 (14)
C8—C7—C12	121.9 (3)	O4—P1—O1	110.20 (12)
C8—C7—N3	119.8 (3)	O2—P1—O1	103.14 (11)
C12—C7—N3	118.2 (3)	O3—P1—O1	108.14 (12)
C7—C8—C9	119.8 (4)		
			
C6—C1—C2—C3	−0.1 (5)	C12—C7—C8—C9	0.4 (5)
N1—C1—C2—C3	−177.0 (3)	N3—C7—C8—C9	−176.2 (3)
C1—C2—C3—C4	0.5 (5)	C7—C8—C9—C10	−0.5 (5)
C2—C3—C4—C5	−1.1 (6)	C8—C9—C10—C11	0.0 (6)
C3—C4—C5—C6	1.5 (6)	C9—C10—C11—C12	0.7 (6)
C2—C1—C6—N2	−175.3 (3)	C10—C11—C12—N4	175.2 (3)
N1—C1—C6—N2	1.4 (4)	C10—C11—C12—C7	−0.8 (5)
C2—C1—C6—C5	0.4 (4)	C8—C7—C12—N4	−175.7 (3)
N1—C1—C6—C5	177.2 (3)	N3—C7—C12—N4	0.9 (4)
C4—C5—C6—C1	−1.1 (5)	C8—C7—C12—C11	0.3 (4)
C4—C5—C6—N2	174.7 (3)	N3—C7—C12—C11	176.9 (2)

### Hydrogen-bond geometry (Å, º)

**Table:**

*D*—H···*A*	*D*—H	H···*A*	*D*···*A*	*D*—H···*A*
O1—H1*D*···O2^i^	0.85 (1)	1.65 (1)	2.470 (3)	164 (4)
N3—H3*A*···O3^i^	0.90 (2)	2.06 (2)	2.928 (3)	160 (3)
N1—H1*A*···O4^ii^	0.92 (2)	1.81 (2)	2.720 (3)	171 (3)
N1—H1*C*···O4^iii^	0.93 (2)	2.02 (2)	2.953 (3)	179 (3)
N2—H2*A*···O4^iii^	0.92 (2)	1.99 (2)	2.904 (4)	170 (3)
N4—H4*A*···O4^iv^	0.88 (2)	2.45 (3)	3.188 (4)	142 (3)
N3—H3*B*···O3^iv^	0.91 (2)	1.87 (2)	2.740 (3)	159 (3)
N3—H3*C*···O3	0.91 (2)	1.87 (2)	2.778 (3)	176 (3)
N1—H1*B*···O2	0.92 (2)	1.83 (2)	2.734 (3)	169 (3)
N4—H4*B*···N2^v^	0.89 (2)	2.33 (2)	3.210 (4)	172 (3)

Symmetry codes: (i) *x*+1, *y*, *z*; (ii) −*x*+1, −*y*+2, −*z*+2; (iii) −*x*, −*y*+2, −*z*+2; (iv) −*x*+1, −*y*+1, −*z*+2; (v) *x*, *y*−1, *z*.
